# The attitude of Belgian social insurance physicians towards evidence-based practice and clinical practice guidelines

**DOI:** 10.1186/1471-2296-10-64

**Published:** 2009-09-09

**Authors:** Annemie Heselmans, Peter Donceel, Bert Aertgeerts, Stijn Van de Velde, Dirk Ramaekers

**Affiliations:** 1 School of Public Health, Katholieke Universiteit Leuven, Leuven, Belgium; 2 Belgian Branch of the Cochrane Collaboration, Belgian Centre for Evidence-Based Medicine, Leuven, Belgium; 3 ZNA Hospital Network Antwerp, Antwerp, Belgium

## Abstract

**Background:**

Evidence-based medicine has broadened its scope and is starting to reach insurance medicine. Although still in its initial stages, physicians in the area of insurance medicine should keep up-to-date with the evidence on various diseases in order to correctly assess disability and to give appropriate advice about health care reimbursement. In order to explore future opportunities of evidence-based medicine to improve daily insurance medicine, there is a need for qualitative studies to better understand insurance physicians' perceptions of EBM. The present study was designed to identify the attitude of insurance physicians towards evidence-based medicine and clinical practice guidelines, and to determine their ability to access, retrieve and appraise the health evidence and the barriers for applying evidence to practice.

**Methods:**

A cross-sectional survey study was carried out among all Dutch-speaking insurance physicians employed at one of the six Belgian social insurance sickness funds and at the National Institute of Disability and Health care Insurance (n = 224). Chi-square tests were used to compare nominal and ordinal variables. Student's t-tests, ANOVA, Mann-Whitney and Kruskal-Wallis were used to compare means of continuous variables for different groups.

**Results:**

The response rate was 48.7%. The majority of respondents were positive towards evidence-based medicine and clinical practice guidelines. Their knowledge of EBM was rather poor. Perceived barriers for applying evidence to practice were mainly time and lack of EBM skills.

**Conclusion:**

Although the majority of physicians were positive towards EBM and welcomed more guidelines, the use of evidence and clinical practice guidelines in insurance medicine is low at present. It is in the first place important to eradicate the perceived inertia which limits the use of EBM and to further investigate the EBM principles in the context of insurance medicine. Available high-quality evidence-based resources (at the moment mainly originating from other medical fields) need to be structured in a way that is useful for insurance physicians and global access to this information needs to be ensured.

## Background

Evidence-based medicine (EBM) has been coined as the conscientious, explicit and judicious use of current best evidence in making decisions about the care of individual patients [1]. Evidence-based medicine has broadened its scope to include areas other than clinical disciplines and is starting to reach the field of insurance medicine.

The setting of the insurance physician differs from that of clinical care. Social insurance physicians in Belgium work for one of the six Belgian social insurance sickness funds or for the government and are not legally allowed to combine this with their own curative practice. Social insurance physicians working for the Belgian Sickness and Health care Insurance evaluate a patient's ability to work and a patient's entitlement to sickness benefits from the social security system. They also assess the legal criteria for reimbursement of expensive health care costs. Medical inspectors of the National Institute of Disability and Health care Insurance (NIDHI) furthermore investigate misuse and defrauding of the reimbursement system by health care providers. The academic training program in Belgian insurance medicine was re-organized in 2005-2006. A Master's in insurance medicine and medico-legal expertise can now be obtained after following a 2-year academic training course [2].

Although the application of evidence-based medicine to the practice of insurance medicine is still in its initial stages, it is clear that physicians in the area of insurance medicine should keep up-to-date with the evidence on various diseases in order to correctly assess disability and to give appropriate advice about health care reimbursement [3]. Although it is true that social insurance and workers' compensation legislation differ between countries [4], evidence about, for instance, the course of a disease and its impairments can be useful in determining disability pension outcomes and incapacity benefits more objectively. Research in insurance medicine is growing, and guidelines developed in other countries can be informative and useful for insurance physicians in Belgium.

Kok R. et al [3] have already evaluated a workshop on EBM for Dutch social insurance physicians who perform disability evaluations. Except for this manuscript, no studies were found relating to the evaluation of the principles of evidence-based medicine in insurance medicine, neither about insurance physicians' attitudes towards or perceptions of evidence-based medicine.

In order to explore future opportunities in evidence-based medicine to improve daily insurance medicine, there is a need for qualitative studies to better understand insurance physicians' perceptions towards EBM. In Belgium, it is not known to what extent insurance physicians integrate evidence in their medical decisions and which information services or sources they use. Their ability to access, retrieve and appraise the health evidence is unclear.

The objectives of the study were to gather data and information about the current insurance medicine situation in the Dutch-speaking part of Belgium.

To fulfil the objectives of our study, we composed a questionnaire to identify insurance physicians'

• access and use of information sources

• attitude towards evidence-based medicine

• perceived knowledge about EBM

• views on perceived barriers to practising EBM

• attitude towards clinical practice guidelines (CPG's)

## Methods

### Study population

The study population of this survey were the Dutch-speaking insurance physicians (224 physicians could be reached) employed at one of the six Belgian social insurance sickness funds and the medical inspectors employed at the National Institute of Disability and Health care Insurance. Insurance physicians from private organisations were not included in the study. 164 physicians employed at the Belgian social insurance sickness funds and 60 physicians employed at the NIDHI were invited to participate.

### Questionnaire development

The development of an instrument to assess Belgian insurance physicians' attitudes towards evidence-based medicine and clinical practice guidelines was based on a comprehensive literature review of publications of existing questionnaires.

This literature review led us to base our questionnaire on that of McColl et al [5]. The survey was adapted to the Belgian setting of insurance medicine and was complemented with questions deemed important for our research population [6,7].

The questionnaire was reviewed by a panel of 8 experts and discussed in a validation meeting to establish content validity. The revised questionnaire was piloted in a group of 5 insurance physicians and modified based on the feedback we received from this group (see Additional file 1).

### Survey administration

The questionnaire was administered online to 224 insurance physicians from mid-October through November 2007. Approval was obtained from the board of each health insurance organization and the NIDHI; physicians were approached by e-mail via their health insurance organisation or via the NIDHI. The survey was anonymous; the whole population sample received two reminders to fill in the survey.

### Data analysis

We used SPSS 14.0 and StatXact 7 for statistical analyses. Descriptive statistics and graphical displays were conducted to describe the sample population. Frequency tables and bar charts were utilized to describe nominal and ordinal variables; continuous variables were described using distributions, means, medians, standard deviations and range.

Chi-square tests were used to compare nominal and ordinal variables. Exact tests were used for those variables that still had sparse responses after combining and recoding responses. Student's t-tests, ANOVA, Mann-Whitney and Kruskal-Wallis were used to compare means of continuous variables for different groups.

We calculated a combined EBM knowledge score and a combined attitude score towards evidence-based medicine and clinical practice guidelines. The combined attitude score was computed by calculating the sum of the positive attitude statements and the reversed answers of the negative attitude statements, excluding the statements which were neither positive nor negative. Pearson's and Spearman's rank correlations coefficients were determined between variables.

All statistics were performed using a 2-sided test and a significance level of 0.05. Bonferroni adjustment was applied for the correlations to correct for multiple comparisons.

## Results

### Study population

A response rate of 48.7% was obtained. 109 questionnaires were returned, of which 4 were not included in analysis: 2 were duplicates, 1 was not usable because of more than 75% missing values in the Likert scales and 1 did not correspond to the inclusion criteria of the population. We finally included 105 questionnaires in data analysis.

The socio-demographic characteristics of the respondents and the whole population of physicians are summarized in table 1 in appendix. The majority of respondents were men, 42.9% represented the 45- to 54-years old group and 35.2% represented the +55 years old group. When comparing these demographic characteristics with the age and sex of the whole population of physicians we can conclude that an equal percentage of men and women participated in the study. The response rate in the 45- to 54- years old group was slightly higher than in the +55 years old group; the percentage of respondents in the other age groups corresponded with the age distribution of physicians in the whole population (see table 1).

**Table 1:** Socio-demographic characteristics of the respondents

***Characteristics***	**Respondents in analysis**	**Total group of physicians**
	**N = 105**	**N = 224**
	**n (%)**	**%**

Sex		
Female	35 (33.3)	32.6
Male	70 (66.7)	67.4

Age		
25-34	3 (2.9)	2.2
34-44	20 (19.0)	19.6
45-54	45 (42.9)	36.4
55+	37 (35.2)	41.8

Employment		
Physician at a health insurance organisation	77 (73.3)	
Medical inspector at the NIDHI	27 (25.7)	
Other	1 (1.0)	

Full-time	93 (88.6)	
Part-time	12 (11.4)	

Use of Electronic Medical Records		
Yes	22 (21.0)	
No	83 (79.0)	

Degree of use of Electronic Medical Records		
Complete	5 (4.8)	
Partial	17 (16.2)	

### Access and use of information sources

The most frequently used information sources were specifically defined journals (reported by 66.7% of physicians), followed by general electronic databases such as Medline (reported by 37.1% of physicians) and the internet in general (mostly searched via Google) (reported by 32.4% of physicians). The most frequently reported journal and website were the 'Tijdschrift voor Geneeskunde' (a Belgian general medical journal in Dutch) and the BCFI website (a Belgian website with pharmacological information).

No-one reported using bibliographic databases daily; the majority indicated using bibliographic databases several times a month (34.3%) or several times a year (33.3%). There was no statistically significant relationship between having access to the whole text of articles and reading the whole text of articles.

62,9% physicians had personal access to electronic databases which seemed to influence the frequency of use (p < 0.001). Those who had followed an EBM course (50.5%), were not more likely to have access to electronic biomedical databases at home or during consultation. The most common reason for searching the literature was 'to support my medical decisions' (reported by 70.5% of physicians) and to keep up-to-date (61.9%).

Further information about the access and use of information sources can be found in table 2.

**Table 2:** Access to information sources

**N = 105**				
		**Yes(%)**	**No(%)**	

Own Documentation Service		47.6	52.4	

Access to electronic biomedical databases		62.9	37.1	

Access to medical or scientific journals on paper		70.5	29.5	

	**At home(%)**	**During consultation(%)**	**At the office(%)**	**At the library(%)**

Access to the internet	92.4	55.2	86.7	7.6

Access to electronic biomedical databases	37.1	23.8	52.4	5.7

% access to full texts of electronic databases	28.2	28.0	32.7	83.3

Access to medical or scientific journals on paper	41.9	10.5	45.7	14.3

### Attitudes towards evidence-based medicine and clinical practice guidelines

56.2% had read about evidence-based medicine, 50.5% had attended an EBM course and 18.1% became familiar with EBM during their basic medical training.

**Figure 1:**
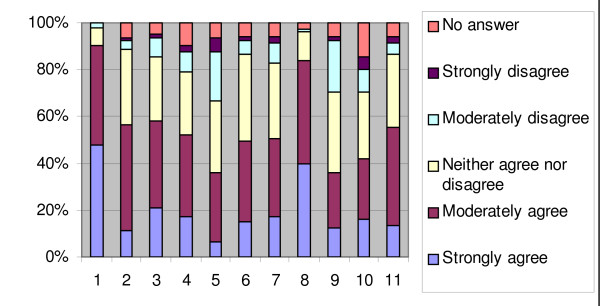
**Percentage of responses for the attitude towards EBM (N = 105)**. (1) My attitude towards evidence-based medicine is positive (2) The attitude of my colleagues is positive towards EBM (3) EBM is useful in daily practice (4) I try to base my medical decisions and/or advice during consultation on evidence (5) I find it difficult to base my medical advice on evidence (6) The use of EBM could lead to better medical decisions and advice (7) Practising EBM involves a decrease in costs (8) There is a lack of scientific studies in insurance medicine (9) Other things are more important than the evidence in the practice of insurance medicine (10) The use of EBM during consultation involves an extra workload (11) I have confidence in the evidence-based value of daily information sources in the field

**Figure 2:**
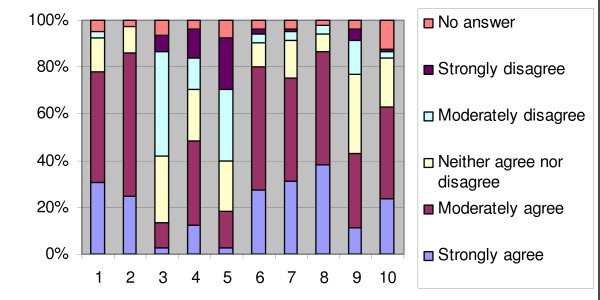
**Percentage of responses for the attitude towards CPG's (N = 105)**. (1) My attitude towards clinical practice guidelines is positive (2) I perceive guidelines as a useful information source (3) Clinical guidelines are mostly not applicable in daily practice (4) The opinion of experts is the most important element during guideline development (5) The integration of guidelines into practice restricts my therapeutic freedom (6) It is important that guidelines are based on research evidence (7) The development of more clinical practice guidelines is welcome (8) The use of guidelines could lead to better quality of care (9) Guidelines are implemented in view of a decrease in financial costs (10) I would like to have electronic recommendations available during consultation

Figure 1 and 2 show the responding physicians' attitudes towards EBM and CPG's.

Physicians were mainly positive about EBM (90.5%) and CPG's (78.1%).

There was an average of 30% neutral responses for each of the EBM attitude statements except for the statements 'My attitude towards evidence-based medicine is positive' and 'There is a lack of scientific studies in insurance medicine'. 83.8% of respondents agreed that there is a lack of scientific studies in insurance medicine.

Differences of opinion existed about the difficulty of basing their medical advice on evidence (36.2% agreed, while 26.7% disagreed). 36.2% of respondents found that 'Other things are more important than the evidence in daily insurance practice' while 23.8% did not agree with this statement.

Only a very small percentage of respondents would not welcome the development of more clinical practice guidelines (4.8%) or expressed a negative attitude towards the use of electronic recommendations during their consultations (3.9%). No-one disagreed with the statement that guidelines are a useful information source. A difference of opinion existed about the importance of the opinion of experts during guideline development. 48.6% found the opinion of experts the most important element during guideline development while 31.7% did not. 80% of respondents agreed that it is important to base guidelines on research evidence and 42.8% believed that guidelines are mainly implemented for financial reasons.

The general attitude score towards EBM and CPG's did not differ between physicians with different demographic characteristics (age, sex, employment).

No significant relationship existed between the frequency of use of electronic biomedical databases and the mean general attitude towards EBM. However, respondents with a higher mean general attitude score indicated that the conclusions of the literature did influence their practice more often (p = 0.002). Access to electronic biomedical databases at home and at the office appeared to be positively correlated to the frequency of use of electronic databases (p < 0.001 for each).

Physicians who agreed that the lack of evidence is a potential barrier for applying evidence to practice were more convinced that the opinion of experts is the most important element during guideline development, (p = 0.002). Furthermore, the group that indicated lack of evidence as a potential barrier had higher mean attitude scores towards CPG's in general (p = 0.008).

### EBM skills

**Figure 3:**
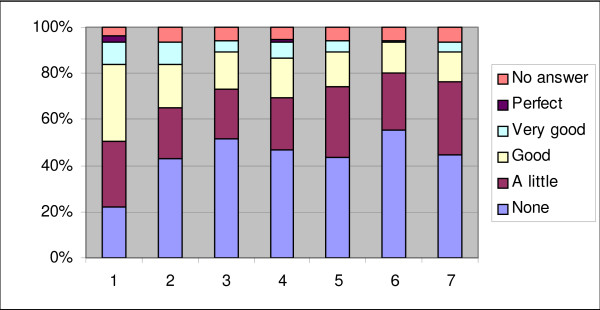
**Percentage of responses for perceived EBM skills (N = 105)**. (1) The ability to search fluently with PubMed or another search engine (2) The use of MeSH terms (3) The ability to formulate a PICO question (4) The use of methodological filters when searching for evidence (5) The ability to recognise potential bias in research designs (6) The use of checklists to evaluate the quality of study designs (7) The ability to interpret research results (e.g. NNT, relative risk reduction, odds ratio, etc)

45.7% of respondents were skilful in searching the literature (good to perfect). 21% of respondents had good to perfect knowledge of formulating a PICO question, 28.5% of physicians were familiar with MesH terms and 23.8% with the use of methodological filters. 20% of respondents had critical appraisal skills, and the perceived ability to interpret statistics ranged from good to perfect in 17.1% of individuals. (Figure 3 gives the percentage of responses for perceived EBM skills)

The respondents with a higher EBM knowledge score also reported using electronic biomedical databases more frequently (p < 0.001). The mean EBM knowledge score appeared to be higher in the group reading literature for research purposes than in the group that did not (p = 0.005). Marginally statistically significant differences existed in general EBM knowledge scores among the different levels of familiarity with EBM (p = 0.05).

Respondents' self-assessed knowledge scores ranged from 0 to 21 out of a possible 28, with a mean of 6.01 ± 5.7. Physicians representing the age group 25-34 years scored the highest amongst all the groups.

### Barriers for the use of EBM

**Figure 4:**
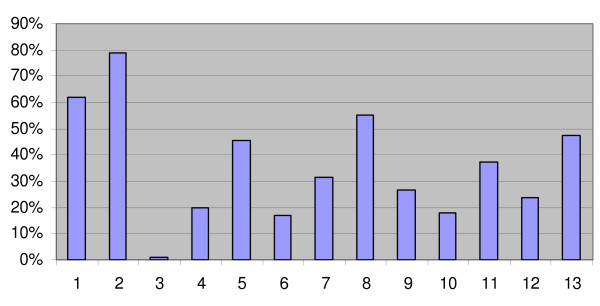
**Potential barriers for applying evidence to practice (N = 105)**. (1) Time (2) EBM skills (3) Concern about losing professional autonomy (4) Lack of support from top management (5) No control over the practice of evidence (6) The pressure to do the same as colleagues (7) Lack of resources (8) Social factors and legislation restrict the usefulness of evidence (9) Lack of financial incentives (10) Evidence different from professional value (11) Lack of evidence (12) Lack of clear presentation of evidence (13) Evidence too difficult/theoretical to apply to practice

Individual barriers were cited most in the list, more specifically EBM skills (79.0%) and time (61.9%). Other frequently reported barriers were the fact that social factors and legislation restrict the usefulness of evidence, (55.2%) the fact that there is no control over the practice of evidence, (45.7%) and that the evidence is too difficult/theoretical to apply to practice (47.6%). Figure 4 summarizes the percentage of responses for each category.

## Discussion

This cross-sectional survey is the first to study the attitude and perceptions of insurance physicians towards evidence-based medicine and clinical practice guidelines. We performed the same study in a group of Belgian occupational health physicians [8] and will rely on the results of this parallel study and results from foreign studies in other medical fields for comparisons.

The response rate was average but acceptable, (48.7%) probably due to the fact that insurance physicians are not as familiar with evidence-based medicine. Further qualitative research would be a good complement to this study to determine the possible reasons for this low response rate and for validating the results. Despite the fact that we invited all physicians employed at public health insurance organizations or the NIDHI, it may be possible that the results are not representative for the whole population of insurance physicians. The survey was self-administered; physicians who are more positive or more familiar with EBM were probably more inclined to fill in the questionnaire. The category of insurance physicians privately employed were not part of our population. We are aware that this group could have had different opinions about EBM and clinical practice guidelines. The EBM knowledge questions did not represent actual knowledge but perceived knowledge which had its limitations and could be biased.

The reliability of the questionnaire was not quantified but logic checks were included. However, the logic checks gave no satisfactory results, probably because a relatively large share of physicians had no strong opinion about EBM and CPG's, supported by the relatively high percentage of neutral responses in the Likert scales of the attitude questions. Despite this, the majority of insurance physicians were still positive about EBM and CPG's, which echoes earlier studies [5,9] and the results of our parallel study in the group of Belgian occupational health physicians [8]. A positive correlation existed between the general attitude towards EBM and the general attitude towards clinical practice guidelines (rho = 0.396 - p = 0.05). No differences in attitude were observed between insurance physicians and medical inspectors of the NIDHI.

No relationship was observed between the general attitude towards EBM and general EBM knowledge. Better knowledge of EBM did not increase the awareness of its importance, supported by the systematic review of Coomarasamy et al [10]. 18.1% became familiar with EBM during their basic training, all of them except one are older than 34 years while the EBM course was only recently integrated in the basic curriculum. These results could indicate that respondents may have had different interpretations of the meaning of an EBM course or the meaning of EBM itself.

45.7% of physicians perceived their searching skills as good to perfect while roughly the same percentage could not formulate a PICO question (51.4% had none to a little knowledge), were not familiar with MeSH terms (42.9%), and could not use methodological filters (46.7%), etc. It is not clear whether they considered their searching skills to be part of their EBM skills or not. The results could be explained by the conclusions of Sackett [1] who maintains that physicians think they are already practising EBM while in reality they are not.

Insurance physicians assess work incapacity and applications for health care reimbursement. For both activities it is important to correctly appreciate the diagnostic and therapeutic choices made by the treating physician. It therefore seems logical that the medical directors of social insurance institutions promote and enhance EBM skills among the social insurance physicians. Our results demonstrate that there is still a great need both for training in EBM and implementation of EBM in practice.

Personal characteristics of the respondents were not correlated to attitude and knowledge of EBM. The use of EBM is not related to age but rather personal conviction and practical possibilities.

Lack of time and lack of EBM were the most important barriers which echoes earlier studies [5,11]. The reported barrier in terms of lack of evidence is not typical for all medical specialities but was also observed in our parallel study [8]. Perceived lack of evidence was expected in this population given the difference in social insurance and workers' compensation legislation between countries. Although research is growing in insurance medicine, this perception could be partly solved by informing physicians of existing evidence in other medical fields which could be used to support their disability evaluations. It is remarkable that 55.2% of physicians indicated legal factors as a potential barrier. The tasks of the insurance physician are determined by Belgian legislation. The characteristics of the tasks of the insurance physician and the legal criteria leave little room for interpretation which is perceived as a potential barrier by many physicians. However, this is only partly true, as an insurance physician has to be able to correctly assess each medical condition based on good evidence.

The reported high impact of the conclusions of the literature on daily work (electronic as well as on paper) is somewhat in contrast to the low frequency of use of electronic databases. Roughly 10% admitted using electronic biomedical databases several times a week while 50% of the respondents reported that they had searched the literature to solve a specific problem for the last time last week. The same discrepancy in results was found in our parallel study in the group of occupational health physicians [8]. The results here led us to presume that sources of literature other than electronic bibliographic databases were used to solve specific medical problems. This is to be expected considering the age of the physicians and the fact that journals were reported as the most important information source.

The majority of physicians gather their information here and there, and the access to high-quality information sources was low. It is possible we may have over- or underestimated the access to information sources because respondents may have interpreted 'access to' as 'awareness of', e.g. no-one reported having access to Bandolier while the information on the Bandolier website is free.

Efforts should be focused on improving personal access to electronic databases and the internet at the location where medical advice is provided; physicians with access should be encouraged to regularly search and use the literature and corresponding electronic databases. Clinical practice guidelines could be a way of making the evidence directly useful for insurance physicians, considering the correlation between the attitude towards CPG's and EBM plus the positive attitude towards CPG's.

## Conclusion

It is hoped that this survey provides further impetus for integrating evidence into the practice of insurance medicine. Although the majority of physicians were positive about EBM and welcomed more guidelines, the use of evidence and clinical practice guidelines in insurance medicine is low at present. It is predominantly important to eradicate perceived inertia which limits the use of EBM and to further investigate the EBM principles in the context of insurance medicine. Existing high-quality evidence-based resources (at the moment mainly originating from other medical fields) need to be structured in a way that is useful for insurance physicians and global access to this information needs to be ensured.

## Competing interests

The authors declare that they have no competing interests.

## Authors' contributions

All authors were involved in various stages of the study design. AH implemented the study, analysed the data and wrote the manuscript. DR supervised the project. DR, PD, BA and SVDV gave methodological advice, commented on subsequent drafts of the paper and reviewed the final text. All authors read and approved the final manuscript.

## Pre-publication history

The pre-publication history for this paper can be accessed here:



## Supplementary Material

Additional file 1 **Questionnaire Insurance Medicine**. Questionnaire used for online survey of insurance physicians' attitude towards evidence-based medicine and clinical practice guidelines, access and use of information sources. Click here for file
