# Supplementary material for: The attitude of Belgian social insurance physicians towards evidence-based practice and clinical practice guidelines
Source: BMC Fam Pract. 2009 Sep 9;10:64. doi: 10.1186/1471-2296-10-64 (PMC2745368; doi:10.1186/1471-2296-10-64)
Supplement: Additional file 1 — Questionnaire Insurance Medicine. Questionnaire used for online survey of insurance physicians' attitude towards evidence-based medicine and clinical practice guidelines, access and use of information sources. [file 1471-2296-10-64-S1.doc]

**Questionnaire Insurance Medicine**

**Personal data**

**Sex**

🔾 Male 🔾 Female

**Year of Birth_____**

**Employment**

🔾 Insurance physician employed at a social insurance sickness fund

🔾 Insurance physician employed at the NIDHI

🔾 Others:____________________________

**Province____________________________**

**I work as insurance physician**

🔾 Full-time

🔾 Part-time

**My main activity is___________________**

**Do you use Electronic Medical Records during the disability assessment consultation?**

🔾 Yes 🔾 No

**If yes, please specify the use of Electronic Medical Records**

🔾 Complete (for all patients’ information)
🔾 Partial (a part of the patients’ information on paper)

**Access to information sources**

**Are you supported by a documentation or a study department to provide literature if needed?**

🔾 Yes 🔾 No

**Where do you have access to the internet?**

🞏 At home

🞏 At my place of consultation

🞏 At my office

🞏 At the (medical) library

🞏 At other places, please specify__________________________________

**Do you have personal access to electronic biomedical databases such as Medline?**

🔾 Yes 🔾 No

**Where do you have access to these electronic biomedical databases?**

🞏 At home

🞏 At my place of consultation

🞏 At my office

🞏 At the (medical) library

🞏 At other places, please specify__________________________________

**Which percentage of your working hours do you have access to these electronic biomedical databases?**

_____%

**Do you have personal access to printed medical journals (on paper)?**

🔾 Yes 🔾 No

**Where do you have access to these journals?**

🞏 At home

🞏 At my place of consultation

🞏 At my office

🞏 At the (medical) library

🞏 At other places, please specify__________________________________

**To which of the following journals do you have full access (electronically or on paper)?**

🞏 Annals of Internal Medicine

🞏 Bandolier

🞏 BMJ

🞏 The Cochrane Library

🞏 JAMA

🞏 Lancet

🞏 Minerva

🞏 New England Journal of Medicine

🞏 Tijdschrift voor Bedrijfs- en verzekeringsgeneeskunde

**Use of information sources**

**Please note your three most important information sources (1 = most important)**

1__________________________________________________________________________

2__________________________________________________________________________

3__________________________________________________________________________

**How much time do you spend on average a week on searching and reading the literature?**

On average: _____hours _____ minutes

**How many times do you use medical bibliographic databases such as Medline for searching scientific medical literature?**

🔾 Never

🔾 Several times a year

🔾 Several times a month

🔾 Several times a week

🔾 Several times a day

**For which percentage of searched articles do you read?**

Only the abstracts _____%

The whole text of the article _____%

**What are you using the information for?**

🞏 To keep up-to-date

🞏 In scope of a postgraduate training

🞏 To support my medical decisions

🞏 Out of curiosity

🞏 In scope of scientific research

🞏 Others, please specify___________________________________________________________________

**Do conclusions of the literature influence your practice?**

🔾 Never 🔾 Seldom 🔾 Sometimes 🔾 Often 🔾 Always

**When have you searched the literature for the last time to solve a specific problem?**

🔾 Never

🔾 More than a year ago

🔾 Last year

🔾 Last month

🔾 Last week

**Attitude towards evidence-based medicine**

**To which degree are you familiar with the methods of evidence-based medicine?**

🞏 I read about it

🞏 I became familiar with it during my basic medical training

🞏 I followed an EBM course

🞏 Others, please specify ____________________________________________________

**Please indicate if you (dis)agree with the following attitude statements**

|  | *Strongly agree (%)* | *Moderately agree (%)* | *Neither agree nor disagree (%)* | *Moderately disagree (%)* | *Strongly disagree (%)* |
| --- | --- | --- | --- | --- | --- |
| My attitude towards evidence-based medicine is positive |  |  |  |  |  |
| The attitude of my colleagues towards EBM is positive |  |  |  |  |  |
| EBM is useful in daily practice |  |  |  |  |  |
| I try to rely on evidence for my medical decisions and/or advice during consultations |  |  |  |  |  |
| I find it difficult to rely on evidence for my medical advice |  |  |  |  |  |
| The use of EBM can lead to better medical decisions and advice |  |  |  |  |  |
| The use of EBM decreases costs |  |  |  |  |  |
| There is a lack of scientific studies in insurance medicine |  |  |  |  |  |
| Other things are more important than the evidence in the practice of insurance medicine |  |  |  |  |  |
| The use of EBM during consultation involves an extra workload |  |  |  |  |  |
| I have confidence in the evidence-based value of daily information sources in the field |  |  |  |  |  |

## EBM skills

## With which EBM skills are you familiar

|  | | | | | |
| --- | --- | --- | --- | --- | --- |
|  | *None (%)* | *A little (%)* | *Good (%)* | *Very good (%)* | *Perfect (%)* |
| The ability to search fluently with PubMed or another search engine |  |  |  |  |  |
| The use of MeSH terms |  |  |  |  |  |
| The ability to formulate a PICO question |  |  |  |  |  |
| The use of methodological filters when searching for evidence |  |  |  |  |  |
| The ability to recognise potential bias in research designs |  |  |  |  |  |
| The use of checklists to evaluate the quality of study designs |  |  |  |  |  |
| The ability to interpret research results (e.g. NNT, relative risk reduction, odds ratio, etc) |  |  |  |  |  |

**Barriers for the use of evidence-based medicine**

**Which factors are in your opinion potential barriers for the integration of EBM into practice**

| Barriers |  |
| --- | --- |
| Individual. professional  Time  EBM skills  Concern about losing professional autonomy  Others, please specify______________________________________ | 🞏  🞏  🞏  🞏 |
| Social context  Lack of support from top management  No control over the practice of evidence  The pressure to do the same as colleagues  Others, please specify______________________________________ | 🞏  🞏  🞏  🞏 |
| Characteristics of the organisation  Lack of resources  Social factors and legislation restrict the usefulness of evidence  Lack of financial incentives  Others, please specify______________________________________ | 🞏  🞏  🞏  🞏 |
| Characteristics of the evidence  Evidence different from professional value  Lack of evidence  Lack of clear presentation of evidence  Evidence too difficult/theoretical to apply to practice  Others, please specify______________________________________ | 🞏  🞏  🞏  🞏  🞏 |

**Please note your 3 most important barriers (1 = most important)**

1______________________________________________________________________________

2**______________________________________________________________________________**

3______________________________________________________________________________

**Attitude towards clinical practice guidelines**

**Please indicate if you (dis)agree with the following attitude statements**

|  | *Strongly agree (%)* | *Moderately agree (%)* | *Neither agree nor disagree (%)* | *Moderately disagree (%)* | *Strongly disagree (%)* |
| --- | --- | --- | --- | --- | --- |
| My attitude towards clinical practice guidelines is positive |  |  |  |  |  |
| I perceive guidelines as a useful information source |  |  |  |  |  |
| Clinical guidelines are mostly not applicable in daily practice |  |  |  |  |  |
| The opinion of experts is the most important element during guideline development |  |  |  |  |  |
| The integration of guidelines into practice restricts my therapeutic freedom |  |  |  |  |  |
| It is important that guidelines are based on research evidence |  |  |  |  |  |
| The development of more clinical practice guidelines is welcome |  |  |  |  |  |
| The use of guidelines could lead to better quality of care |  |  |  |  |  |
| Guidelines are implemented in view of a decrease in financial costs |  |  |  |  |  |
| I would like to have electronic recommendations available during consultation |  |  |  |  |  |
